# Effects of different postharvest drying processes on flavonoid content and enzymatic activity of *Styphnolobium japonicum* (L.) Schott flowers for industrial and medicinal use

**DOI:** 10.1016/j.heliyon.2024.e35095

**Published:** 2024-07-23

**Authors:** Ya-Feng Zuo, Xin-Qiu Liu, Xiang-Song Meng, Meng-Hu Wang, Jian Tang, Ting-Ting Hu, Wen-Jian Wang, Wei Zhang, De-Ling Wu

**Affiliations:** a School of Chinese Medicine, Bozhou University, Bozhou 236800, China; b Key Laboratory of Chinese Medicine Materials Research of Anhui Higher Education Institutes, Bozhou University, Bozhou 236800, China; c Department of Pharmacy, Bozhou Hospital of Traditional Chinese Medicine, Bozhou 236800, China; d School of Pharmacy, Anhui University of Chinese Medicine, Hefei 230012, China

**Keywords:** *S. japonicum* flower, *S. japonicum* flower bud, Postharvest drying, Rutin, Rutin-hydrolyzing enzyme

## Abstract

Traditionally, fresh *S. japonicum* flowers (SJF) and *S. japonicum* flowers buds (SJFB) are dried prior to further processing and use. Here, we investigated the ways in which drying techniques, including sun drying (SD), steam drying (STD), microwave drying (MD), hot air drying (HAD, 40 °C, 60 °C, 80 °C, 100 °C), and freeze drying (FD), alter the flavonoid composition of freshly-harvested SJF and SJFB. The flavonoid content of dried samples was determined by Ultra High Performance Liquid Chromatography-Diode Array Detector (UPLC-DAD). Overall, different drying techniques had significantly different effects on the RU content, ranging from 10.63 % (HAD-80 °C) to 34.13 % (HAD-100 °C) in SJF and from 18.91 % (HAD-100 °C) to 29.16 % (HAD-40 °C) and 30.53 % (SD) in SJFB. To clarify the mechanism by which drying affects the RU content of *S. japonicum* flowers, we studied the activity of a rutin-hydrolyzing enzyme (RHE) isolated from SJF and SJFB using multiple separation and assay methods. According to the Sodium Dodecyl Sulfate-Polyacrylamide Gel Electrophoresis (SDS-PAGE) results, the apparent molecular weight of the purified RHE was approximately 38 kDa. According to UPLC-DAD, RHE catalyzes the production of quercetin (QU) from rutin (RU), but not from other flavonoid glycosides. Drying fresh SJF and SJFB at low and high temperatures can inhibit RHE activity and prevent RU hydrolysis. Therefore, subjecting freshly-harvest SJF to HAD-100 °C, and freshly-harvest SJFB to SD or HAD-40 °C, can greatly increase the RU content. In particular, HAD is viable for large-scale application due to its simplicity and industrial feasibility.

## 1 Introduction

The flowers and floral buds of the Japanese pagoda tree (*Styphnolobium japonicum* (L.) Schott; Fabaceae) have been utilized for centuries in traditional Chinese medicine to address hematochezia, intestinal hemorrhage, arteriosclerosis, dysentery, headache, dizziness, and pyodermas [[1], [2], [3]]. The medicinal properties of *S. japonicum* flowers are mainly attributed to flavonoids such as kaempferol (KA), quercetin (QU), and rutin (RU), which are considered the main active ingredients and quality indicators of *S. japonicum* flowers [4,5]*.* Flavonoids are natural organic compounds widely found in plants, with a variety of biological activities such as antioxidant, anti-inflammatory, antimutagenic and anticancer. In recent years, with the in-depth research on flavonoids, it has been found that they have a wide range of applications in the fields of nutraceuticals, pharmaceuticals and medicinal uses [[6], [7], [8]]. A growing body of literature suggests that *S. japonicum* flowers and RU exhibit anti-tumor, anti-inflammatory, and antioxidant effects, as well as confer protection against cardiovascular and cerebrovascular diseases [[9], [10], [11], [12], [13]]. Such findings highlight the utility of *S. japonicum* flowers to the pharmaceutical and chemical industries.

Fresh *S. japonicum* flowers are typically air-dried following harvest. However, because photosynthesis shuts down after harvesting, respiration becomes the main metabolic process, resulting in the catabolic consumption of phytochemicals. In addition, some enzymes remain active during the drying process, resulting in the decomposition of bioactive substances and, ultimately, loss of therapeutic efficacy [14]. Because of this, the drying process requires optimization to preserve the quality of medicinal materials. Historically, *S. japonicum* flowers and floral buds are collected in summer and sun-dried [15]. Although sun drying is traditional, it may not be the most suitable method for drying both SJF and SJFB due to the characteristic properties of each material. The efficiency of sun drying can also be affected by weather, necessitating the use of industrial techniques such as microwave drying and hot air drying. Zhen et al. [16] found that steaming followed by hot air drying or freeze drying increased the polyphenol content and antioxidant activity of *Gastrodia elata* compared with other drying methods. Tsurunaga et al. [17] investigated the effects of steaming time and drying temperature on Genova's antioxidant composition and properties, color and aroma, and found that freeze drying without steaming was the best method for retaining Genova's three main aroma components. Xue et al. [18] investigated the effects of different drying methods on the aroma and flavor of *Bletilla striata* Scented Tea. Compared with other drying methods, freeze drying method could retain flavonoids, polysaccharides and phenolic components well, and also had better antioxidant and antibacterial properties.

To address this knowledge gap, we sought to investigate the influence and mechanism of various drying methods, including sun drying (SD), hot air drying (HAD), microwave drying (MD), steam drying (STD), and freeze drying (FD), on the major bioactive constituents of fresh post-harvest SJF and SJFB.

## 2 Materials and methods

### 2.1 Sample collection and processing

Six kilograms of fresh SJF and SJFB were harvested in July 2021 from Bozhou, Anhui Province, China. One portion of the freshly-harvested SJF and SJFB was flash frozen in liquid nitrogen and subsequently reserved at −80 °C. The remaining was set aside for SD, HAD, MD, STD, and FD. For SD, the freshly-harvested SJF and SJFB were placed on a ceramic plate and dried in direct sunlight. For HAD, the freshly-harvested SJF and SJFB were dried in an electrically-heated thermostatic drying oven (101-2 ES, Shanghai Keheng Industrial Development Co., Ltd., China) at different temperatures (40 °C, 60 °C, 80 °C, 100 °C). For FD, the freshly-harvested SJF and SJFB were first frozen at −40 °C for 24 h and then lyophilized (SCIENTZ-10N/A, Ningbo Xinzhi Biotechnology Co., Ltd., China). For MD, the freshly-harvested SJF and SJFB were evenly spread into a thin layer and microwaved (P70F23P-G5, Guangdong Grantham Microwave Household Appliance Manufacturing Co., Ltd., China) at 0.7 kW for 3 min, and subsequently arranged in a 60 °C oven to finish drying. For STD, the freshly-harvested SJF and SJFB were treated with saturated steam (GZ528, JoYoung Co., Ltd., China) at 100 °C for 3 min, and then placed in a 60 °C oven to finish drying. All samples were dried to a moisture content of less than 11.0 % [19], pulverized, and stored in a dry ventilated location [20].

### 2.2 Extraction of flavonoids

Floral samples were extracted according to a previously-published method [21], with modifications. Specifically, 0.5 g of each sample was placed in a stoppered conical flask, to which 8 mL of 70 % ethanol was added. Extraction (Ultrasonic Cleaner JK-2000DB, 40 kHz, 240 W, Hefei Jinnik Machinery Manufacturing Co., Ltd., China) was performed at 60 °C for 30 min. Following filtration through a 0.22 μm membrane, the flavonoids were detected using UPLC-DAD.

### 2.3 Determination of flavonoid contents

**Fig. 1:**
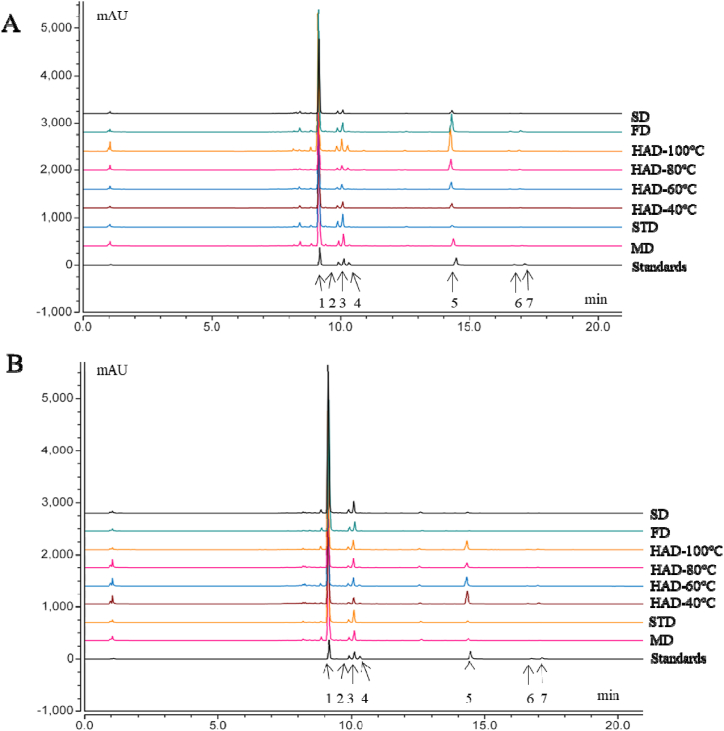
UPLC-DAD chromatograms of mixed standards and *S. japonicum* flowers (A) and floral buds (B) subjected to microwave drying (MD), steam drying (STD), hot air drying (HAD), freeze drying (FD), and sun-drying (SD). 1-rutin (RU), 2-nicotiflorin (NI), 3-narcissoside (NA), 4-astragalin (AS), 5-quercetin (QU), 6-kaempferol (KA), 7-isorhamnetin (IS).

The contents of seven detected flavonoids were determined with UPLC-DAD (Fig. 1A and B), which was performed on an UltiMate 3000 Rapid Separation (RS) system (Thermo Fisher Scientific, Suzhou, China). Separation was carried out at 30 °C using an Accucore-C_18_ column (2.6 μm, 2.1 mm × 100 mm) (Thermo Fisher Scientific, Suzhou, China), with a 0.2 mL/min constant flow rate. The elution gradient was as follows: 0–2 min, 5 % A; 2–4 min, 5–20 % A; 4–8 min, 20 % A; 8–12 min, 20–32 % A; 12–20 min, 32–35 % A; 20–24 min, 35-5 % A; 24–28 min, 5 % A; 28–30 min, 5–90 % A; 30–42min, 90 %A; 42–45min, 90-5 %A; 45–55min, 5 %A. The mobile phase consisted of (A) acetonitrile and (B) water containing 1 % acetic acid. Prior to analyses, all sampled were filtered using a 0.22 μm membrane. The injection volume was 1 μL and the monitored wavelength was 260 nm. Calibration curves were used to quantify the flavonoid content. The regression equations, R^2^ values, and linear ranges can be found in Table 1.

**Table 1:** Regression statistics for seven flavonoids compounds.

Flavonoid	Calibration Curve	Linearity Range (μg/mL)	R^2^
RU (1)	y = 89.267x+1.0851	4.000–3030	0.9986
NI (2)	y = 83.346x+0.1713	2.160–1080	0.9998
NA (3)	y = 99.05x-0.1785	2.032–1016	0.9997
AS (4)	y = 127.39x-0.1031	0.202–1008	0.9999
QU (5)	y = 172.39x-0.5994	2.068–1034	0.9998
KA (6)	y = 177.89x-0.2336	2.030–1015	0.9998
IS (7)	y = 210.28x-0.5333	1.078–215.6	0.9998

### 2.4 Extraction and purification of rutin-hydrolyzing enzyme

The extraction and purification of RHE were carried out at 4 °C. Briefly, 100 g of freshly-harvested *S. japonicum* flowers were mixed in 500 mL of 20 mM acetate buffer (pH 5.0) and allowed to extract at 4 °C for 12 h. The extraction solution was subsequently gauze-filtered to remove residue. The insoluble material was eliminated by centrifugation at 12000*g* for 30 min. Subsequently, the supernatant was slowly mixed with (NH_4_)_2_SO_4_ to a saturation of 80 % and continuously stirred for 4 h. Next, the precipitate was redissolved in 20 mM Tris-HCl buffer (pH 7.0) by centrifugation at 12000*g* and 4 °C for 30 min. The crude enzymes were then dialysed in deionised water at 4 °C for 12 h. The dialysed crude enzyme solution was concentrated with PEG-6000 to a proper volume (5 mL) and stored in a refrigerator at 4 °C until use.

After equilibration with 20 mM acetate buffer (pH 5.0), the resulting crude enzyme (5 mL) was added to a DEAE Celluose-32 column and subsequently eluted with 20 mM acetate buffer (pH 5.0) containing 1 M NaCl. Eppendorf tubes were used to collect unbound and eluted enzyme. For purification, the active enzyme solution was applied to a Sephadex G-100 gel filtration column and eluted at a flow rate of 0.5 mL/min with 20 mM acetate buffer (pH 5.0). Subsequently, the purified RHE was concentrated for further analyses. The concentration of RHE was quantified according to the Bradford technique [22], with bovine serum albumin as the standard. The molecular weight of RHE was estimated by sodium dodecyl sulfate-polyacrylamide gel electrophoresis (SDS-PAGE), according to the Laemmli method [23]. The enzyme molecular weight standards consisted of a mixture of markers supplied by Thermo Fisher Scientific.

### 2.5 Analysis of rutin-hydrolyzing enzyme activity

Thin layer chromatography (TLC) can be used to qualitatively detect QU production in order to intuitively judge the activity of the purified enzyme [24]. According to a previously-published method [21] with slight modifications, the total flavonoid extract was concentrated and lyophilized. Appropriate amounts of RU and total flavonoid (TF) content standards were dissolved in volumetric flasks to prepare substrate solutions at different concentrations: 1.00 mg/mL RU and 0.50 mg/mL TF, respectively. Next, 250 μL of either RU or TF reaction substrate was then mixed with 250 μL of purified RHE. Subsequently, the solutions were incubated at 40 °C for 30 min. The enzyme reaction was interrupted by adding 1500 μL of methanol. As a separate control, purified RHE was inactivated with 1500 μL methanol and then mixed with the substrate solution. The mixture was centrifuged and subjected to C18 column (2.1 mm × 100 mm, 2.6 μm) UPLC-DAD (Thermo Fisher Scientific, China). The amount of QU emitted by RU was used as a substrate to determine the specific activity of RHE. Finally, the reaction solution was analyzed as described in section 2.3.

### 2.6 Statistical analysis

All data are shown as means ± standard deviations. Analysis of variance (ANOVA), performed in SPSS ver. 27.0, was used to determine statistically significant differences. Results were visualized using Origin ver. 2021b.

## 3 Results and discussion

### 3.1 Effect of drying method on flavonoid content in S. japonicum flowers

**Fig. 2:**
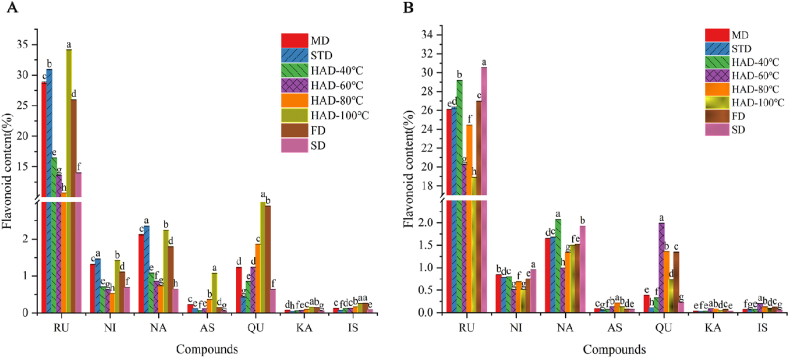
Flavonoid content of SJF (A) and SJFB (B) subjected to MD, STD, HAD, FD, and SD. Statistically significant differences among treatments (*p* < 0.05) are indicated by different lowercase letters.

Drying technique had a significant effect on the flavonoid content of SJF and SJFB (Fig. 2A and B). Specifically, in comparison to freshly-harvested SJF, the RU content decreased in the following order: HAD-100 °C (34.13 ± 0.039 %) > STD (30.91 ± 0.083 %) > MD (28.79 ± 0.254 %) > FD (25.96 ± 0.063 %) > HAD-40 °C (16.41 ± 0.031 %) > SD (14.01 ± 0.025 %) > HAD-60 °C (13.52 ± 0.019 %) > HAD-80 °C (10.63 ± 0.013 %). Overall, the RU content was highest in SJF subjected to HAD-100 °C. The RU content was highest in SJF subjected to HAD-100 °C, STD, MD, and FD (*p* < 0.05). The three flavonoid glycosides nicotiflorin (NI), narcissoside (NA), and astragalin (AS), exhibited similar patterns. In comparison to freshly-harvested SJFB, the RU content decreased in the following order: SD (30.53 ± 0.028 %) > HAD-40 °C (29.16 ± 0.083 %) > FD (26.97 ± 0.060 %) > STD (26.26 ± 0.156 %) > MD (26.09 ± 0.032 %) > HAD-80 °C (24.43 ± 0.012 %) > HAD-60 °C (20.21 ± 0.011 %) > HAD-100 °C (18.91 ± 0.016 %). The RU content was highest in SJFB subjected to SD. Although it is well-established that the drying process can have a significant influence on the bioactive components in the dried material, the precise mechanism by which these components are affected remains unclear [25,26]. For example, SJF and SJFB contain rutin-hydrolyzing enzyme (RHE), which may affect this process [27]. The differences in the appearance and morphology of SJF and SJFB, as well as in the content of hydrolytic enzymes and enzymatic activities contained therein, this may be the reason for the large difference in the active components of rutin and other flavonoids between fresh SJF and SJFB after drying [28]. Again, the two flavonoid glycosides NI, NA exhibited similar patterns. This may be due to the fact that NI, NA and RU all belong to the flavonol group of compounds and they all form brassinosteroid chain glycosides on 3-OH [27].

Both SJF and SJFB contain flavonoid glycoside hydrolases. Drying at either high or low temperatures inactivates or inhibits enzymatic activity, resulting in changes to the flavonoid glycoside content in SJF and SJFB [29]. The hydrolases in SJFB subjected to HAD-100 °C were rapidly inactivated, resulting in minimal RU hydrolysis. Since SJFB is a flower bud, the hydrolases in SJFB were not activated at SD and HAD-40 °C, and RUs were not enzymatically hydrolyzed under these conditions. RU, NA, and NI are glucosides of QU, isorhamnetin (IS), and KA, respectively, and can be hydrolyzed into their corresponding glycosides and disaccharides [21,30]. In SJF and SJFB, drying activates hydrolytic enzyme activity, leading to the hydrolysis of flavonoid glycosides (i.e., RU) into flavonoid glycosides (i.e., QU). However, we observed irregular changes in flavonoid glycoside content between drying treatments, suggesting that these techniques not only affect hydrolase activity, but also affect secondary metabolites directly [31].

In medicinal plants, post-harvest drying and processing preserves product quality, reduces deterioration, and maximizes medicinal value [32]. We observed that different drying techniques resulted in significantly different flavonoid compositions compared to freshly-harvested materials. Specifically, the content of commercially- and medicinally-valuable RU differed significantly among dried materials, ranging from 10.63 % (HAD-80 °C) to 34.13 % (HAD-100 °C) in SJF and from 18.91 % (HAD-100 °C) to 29.16 % (HAD-40 °C) and 30.53 % (SD) in SJFB. Notably, subjecting freshly-harvest SJF to HAD-100 °C, and freshly-harvest SJFB to HAD-40 °C, resulted in increased RU content. This may have resulted from the activity of RHE in freshly-harvested *S. japonicum* flowers*.* However, other flavonoid glycoside hydrolases may be present in *S. japonicum* flowers. Strict substrate specificity related to their catalytic reactivity could explain the observed changes in NA and NI content following drying. Overall, based on these results, HAD appears to be a practical technique to increase the RU content in freshly-harvested *S. japonicum* flowers.

### 3.2 Isolation of rutin-hydrolyzing enzyme

**Fig. 3:**
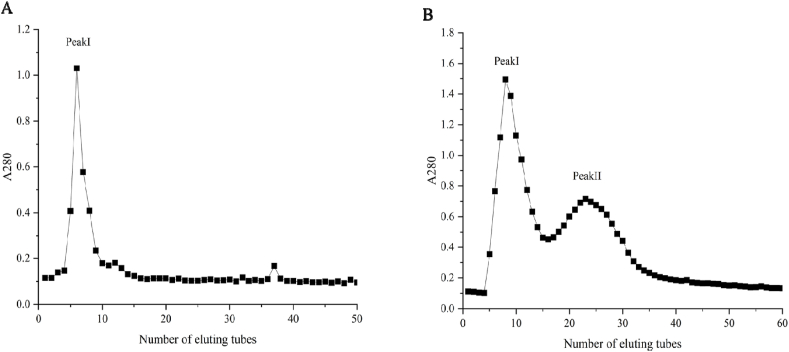
Purification of rutin-hydrolyzing enzyme from *S. japonicum* flowers. Chromatograms obtained using DEAE celluose-32 (A) and Sephadex G-100 (B).

**Fig. 4:**
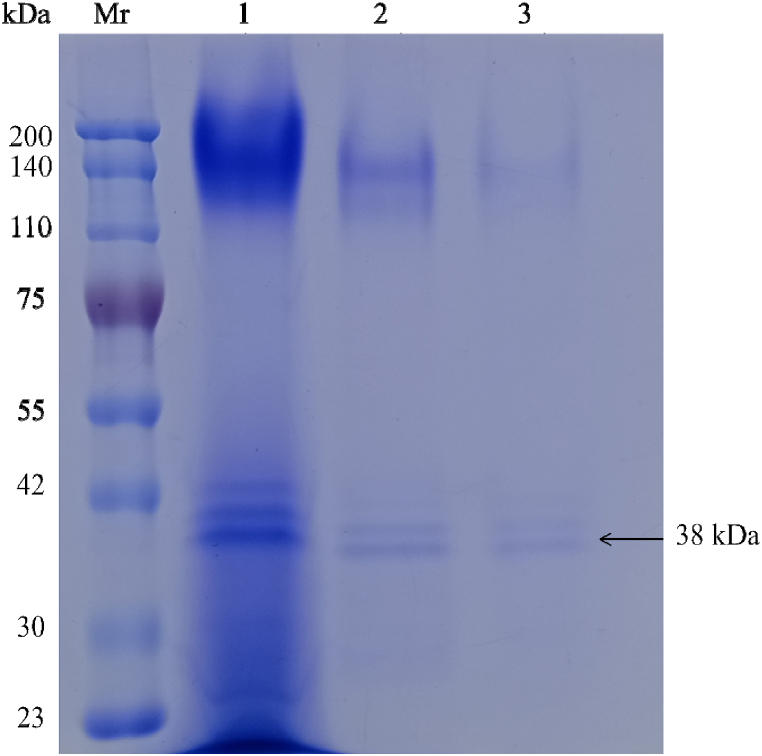
SDS-PAGE performed for each purification step. Lane 1: crude enzyme extract; Lane 2: DEAE-cellulose 32 chromatography activity peak; Lane 3: Sephadex G-100 gel filtration activity peak.

Crude enzymes were dialysed in 20 mM acetate buffer (pH 5.0) and applied to a DEAE-cellulose-32 column, as described in section 2.4. The active enzyme peak was eluted with 18 mL eluent containing 1 M NaCl (Fig. 3A). The peak obtained from the DEAE-cellulose 32 column was further resolved on a Sephadex G-100 gel filtration column with 20 mM acetate buffer (pH 5.0) (Fig. 3B). RU hydrolysis was demonstrated by the enzyme eluted in peak Ⅰ (elution volume: 24.0 ml), and peak Ⅱ did not have RU hydrolysis. Many medicinal plants are rich in flavonoids and their glycosides as well as glycoside hydrolases, and glycoside hydrolases have a certain substrate specificity, and the enzymatic properties and substrate specificity of peak Ⅱ may need to be further investigated [33]. SDS-PAGE showed a highly-purified single enzyme band (Fig. 4, Lane 3). This enzyme was estimated to have a molecular weight of 38 kDa. RHE was efficiently purified (5.66 fold) from ground fresh post-harvest *S. japonicum* flowers. The total recovery was 24.93 % (Table 2). The enzyme purified in our study is considerably different from the glycosidase found in common buckwheat seeds [24,27], suggesting that the RHE purified from *S. japonicum* differs from that in buckwheat.

**Table 2:** Rutin-hydrolyzing enzyme purification and activity.

Step	Volume (mL)	Total Enzyme (mg)	Total Activity (units)	Specific Activity (unit/mg)	Activity Recovery (%)	Purification (fold)
Crude extract	12	14.77	271.16	18.36	100.00	1.00
DEAE celluose-32	16	2.72	192.41	70.74	70.96	3.85
G-100 gel filtration	7	0.65	67.59	103.98	24.93	5.66

### 3.3 Substrate-specificity of rutin-hydrolyzing enzyme

**Fig. 5:**
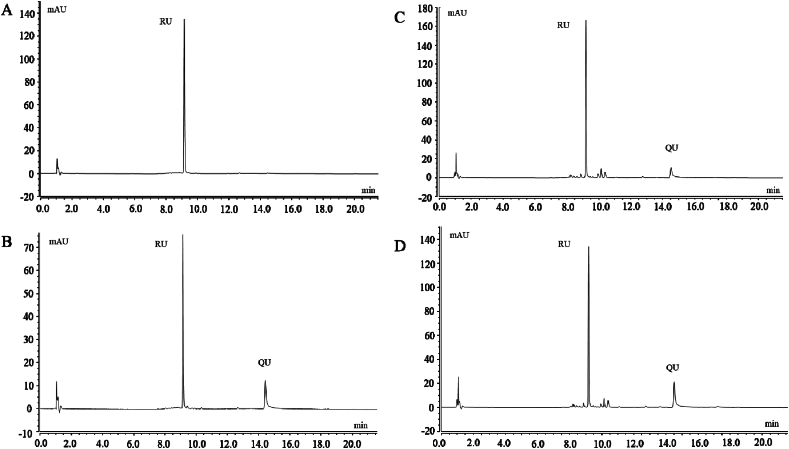
UPLC-DAD of RU content and QU content following TF and RU reaction with (RHE) at 40 °C for 30 min,respectively. (A, C) Reaction with inactivated enzyme. (B, D) Reaction with native enzyme.

We used the partially-purified TF extract (containing NI, NA, AS, and RU) as a substrate for the isolated RHE. The reaction solution was analyzed by UPLC-DAD after 15 min of incubation at 40 °C with either RU or TF and purified RHE (Fig. 5). When RU was used as the reaction substrate, QU was generated and the RU content decreased significantly compared to the control (Fig. 5A and B), suggesting that RHE can effectively convert RU into QU. When TF was used as the reaction substrate, the RU content also decreased significantly compared to the control (Fig. 5C and D). However, a remarkable amount of aglycone-form QU was formed after 30 min of hydrolysis. These results suggest that RU was converted to QU by RHE, whereas NA, NI, and AS did not undergo hydrolysis and remained unchanged. The results shown in Table 3 corroborate this hypothesis, as the content of QU increased and the content of RU decreased due to hydrolysis by RHE. Accordingly, RHE was observed to exhibit strict substrate specificity for QU, but not for any of the other flavonoid glycosides. Other enzymes have been implicated in the production of rutinose and QU from RU, including *Penicillium* enzyme and *Aspergillus* enzyme [34,35]. To our knowledge, no other studies have reported the hydrolysis of RU to form QU in *S. japonicum* flowers. Therefore, our results may form the basis for further research on flavonoid biosynthesis in *S. japonicum*.

**Table 3:** Flavonoid contents in the TF and RU substrate before and after hydrolysis with RHE.

Sample	Content (μg/mg)				
	RU	NI	NA	AS	QU
TF substrate (5 mg)	200	16	5	11	34
Hydrolytic product	149	14	4	11	51
RU substrate (1 mg)	147	–	–	–	–
Hydrolytic product	55	–		–	32

Note: 1-rutin (RU); 2-nicotiflorin (NI); 3-narcissoside (NA); 4-astragalin (AS); 5-quercetin (QU).

## 4 Conclusions

In this work, we evaluated the effects of different drying techniques on the bioactive constituents of freshly-harvested SJF and SJFB. SJF samples subjected to HAD-100 °C exhibited 3.2-fold greater RU content (%) than samples subjected to HAD-80 °C. In addition, SJFB samples subjected to HAD-40 °C exhibited 1.5-fold greater RU content (%) than samples subjected to HAD-100 °C. The three flavonoid glycosides NI, NA, and AS exhibited similar patterns in both SJF and SJFB. These results suggest that HAD is an effective industrial drying treatment to increase the RU content of freshly harvested *S. japonicum* flowers.

The effect of drying technique on the flavonoid content of *S. japonicum* flowers is primarily attributed to the altered activity of hydrolyzing enzymes. Here, we isolated and purified a 38-kDa RHE from *S. japonicum* flowers. This enzyme was observed to exhibit strict substrate specificity for RU, but not for other flavonoid glycosides. The presence of RHE in *S. japonicum* flowers explains the varied effects of different drying methods on the RU content of floral samples. However, other flavonoid glycoside hydrolyzing enzymes may also be present in *S. japonicum* flowers, explaining changes in the NI and NA content in dried samples. Overall, the results of this study help to clarify the effects of different drying treatments on the medicinal quality of botanical materials and will be useful in further optimizing the post-harvest processing of *S. japonicum* flowers.

## Funding statement

This work was supported by the Anhui Provincial Universities Excellent and Top Talents Cultivation Funding Program_Excellent Young Teachers Cultivation Program (YQYB2023064), the 10.13039/501100010814Anhui Provincial Department of Education University Natural Science Research Project (KJ2020A0765, KJ2021A1143, KJ2021A1144, 2022AH052414), the 10.13039/501100020444Bozhou University Traditional Chinese Medicine Team (BYZXKTD202004), the Anhui Province Peak Cultivation Discipline (Traditional Chinese Pharmacology) and the Anhui Red Cross Foundation Chinese Medicine Inheritance Innovation Development Research Program (2022ZYYD29).

## Data availability statement

Data included in article/supp. Material/referenced in article.

## CRediT authorship contribution statement

**Ya-Feng Zuo:** Funding acquisition. **Xin-Qiu Liu:** Validation, Conceptualization. **Xiang-Song Meng:** Validation, Conceptualization. **Jian Tang:** Writing – review & editing, Writing – review & editing. **Hu Ting-Ting:** Writing – original draft, Validation, Project administration, Data curation, Conceptualization. **Wen-Jian Wang:** Writing – review & editing. **Wei Zhang:** Conceptualization. **Wu De-Ling:** Data curation.

## Declaration of competing interest

The authors declare that they have no known competing financial interests or personal relationships that could have appeared to influence the work reported in this paper.
